# New Policies, New Technologies: Modelling the Potential for Improved Smear Microscopy Services in Malawi

**DOI:** 10.1371/journal.pone.0007760

**Published:** 2009-11-10

**Authors:** Andrew Ramsay, Luis E. Cuevas, Catherine J. F. Mundy, Carl-Michael Nathanson, Petros Chirambo, Russell Dacombe, S. Bertel Squire, Felix M. L. Salaniponi, Sera Munthali

**Affiliations:** 1 UNICEF/UNDP/World Bank/WHO Special Programme for Research and Training in Tropical Diseases, World Health Organization, Geneva, Switzerland; 2 Liverpool School of Tropical Medicine, Liverpool, United Kingdom; 3 Management Sciences for Health, Arlington, Virginia, United States of America; 4 REACH Trust, Community Health Sciences Unit, Lilongwe, Malawi; 5 Liverpool Associates in Tropical Health, Liverpool, United Kingdom; 6 National Tuberculosis Control Programme, Ministry of Health, Lilongwe, Malawi; 7 Lilongwe District Health Office, Bwaila Hospital, Lilongwe, Malawi; University of Stellenbosch, South Africa

## Abstract

**Background:**

To quantify the likely impact of recent WHO policy recommendations regarding smear microscopy and the introduction of appropriate low-cost fluorescence microscopy on a) case detection and b) laboratory workload.

**Methodology/Principal Findings:**

An audit of the laboratory register in an urban hospital, Lilongwe, Malawi, and the application of a simple modelling framework. The adoption of the new definition of a smear-positive case could directly increase case detection by up to 28%. Examining Ziehl-Neelsen (ZN) sputum smears for up to 10 minutes before declaring them negative has previously been shown to increase case detection (over and above that gained by the adoption of the new case definition) by 70% compared with examination times in routine practice. Three times the number of staff would be required to adequately examine the current workload of smears using ZN microscopy. Through implementing new policy recommendations and LED-based fluorescence microscopy the current laboratory staff complement could investigate the same number of patients, examining auramine-stained smears to an extent that is equivalent to a 10 minutes ZN smear examination.

**Conclusions/Significance:**

Combined implementation of the new WHO recommendations on smear microscopy and LED-based fluorescence microscopy could result in substantial increases in smear positive case-detection using existing human resources and minimal additional equipment.

## Introduction

There are an estimated 9.3 million new cases of tuberculosis (TB) each year, with the majority occurring in low- and middle-income countries (LMICs) [1]. Most LMICs rely almost entirely on direct sputum smear microscopy (DSSM) for routine TB diagnostic services [2]. The 2008 data indicate that 1.4 million of the TB cases were in people living with HIV/AIDS (PLWHA) who are particularly likely to be paucibacillary or smear-negative [1], [3].

The World Health Organization (WHO) has recently made policy recommendations relevant to sputum microscopy in settings with well-functioning External Quality Assessment (EQA) schemes. The recommendations include lowering the thresholds for: a) classifying a smear as positive from ≥10 Acid Fast Bacilli (AFB)/100 High Power Fields (i.e. 1+ grade) to 1 AFB per smear (ie. including all “scanty” smears), and b) classifying a patient as a smear-positive case (from two positive smears to one) [4]. This has been shown to significantly increase the numbers of cases classified as smear positive [5], [6].

The WHO, recognizing the minimal additional yield provided by the third specimen, has also recommended that, in settings with high laboratory workloads and limited human resources, the minimum number of sputum specimens to be examined in the investigation of suspected PTB could be reduced from three to two [7], [8]. The intention is to improve the quality of smear microscopy (and improve case detection) through alleviating unmanageable workloads and allowing more time for the examination of the two most important specimens.

The sensitivity of smear microscopy is reported to be variable even in epidemiologically similar settings. The amount of time spent examining smears is critical but seldom reported. Adequate time must be invested if paucibacillary cases are to be detected. International guidelines recommend a 5 to 10 minute examination, equivalent to the examination of between 100–300 high-power fields (HPF), before declaring a smear to be negative [2], [9], [10]. Inadequate examination times have been reported in LMICs, often in association with heavy laboratory workloads and shortages of trained personnel [11], [12]. In a district hospital in Cameroon the median duration of smear examination in routine practice was 2 minutes 6 seconds [13]. Even after adoption of the new WHO-approved reduced thresholds for defining a positive smear and a smear positive case, re-examining the same smears for up to 10 minutes increased case detection by 70% [13]. Unfortunately, overwhelming laboratory workloads and a lack of human resources prevent such lengthy examination times in developing countries, particularly in those countries badly affected by the HIV pandemic.

It is recognized that fluorescence microscopy (FM) could also increase case detection and reduce laboratory workloads. A recent systematic review indicates that FM may increase sensitivity of DSSM by around 10%. Perhaps more importantly, the examination of fluorochrome-stained smears can be achieved in 25% of the time taken to examine Ziehl-Neelsen (ZN) stained smears (with comparable specificity) [14], [15]. Thus, the FM equivalent of a 10 minute ZN examination would be just 2.5 minutes - little more than the routine examination times recorded in Cameroon. Until recently the uptake of FM in LMICs has been hampered by the high cost, complexity and poor user-acceptance of conventional FM systems. Low-cost FM based on light-emitting diodes (LED) is now becoming available and this new technology could lead to more widespread use of FM [16], [17].

Both the recent policy changes and LED-FM seem suitable for diagnostic services in Malawi, which have endured long-standing and severe human resource shortages [18], [19]. Sputum smear microscopy workloads in district level laboratories are very high relative to the number of laboratory staff available [11], [19]. The Malawi National TB Control Programme (NTP) has a well-established EQA Scheme [20]. There are however no estimates of the likely benefits to be gained from implementing the new policies and/or new technology, whether alone or in combination. Such estimates would provide useful information to NTPs considering diagnostic retooling [21]–[23].

Retrospective laboratory data from an urban hospital in Malawi was used to model:

1. the increase in smear-positive case detection that might be expected to stem directly from adoption of the WHO-recommended lowered threshold for definition of a positive smear and a smear-positive case;
2. the reduction in laboratory workload that might be expected from adopting a two-specimen strategy for investigating suspected pulmonary TB and
3. the potential for examining ZN stained sputum smears for the recommended 10 minutes (or the LED-FM equivalent) with the existing designated laboratory staff.

This is the first attempt to model the potential impact of these interventions that has been reported as far as we are aware.

## Materials and Methods

This retrospective analysis used data from the laboratory register of Bwaila Hospital in Lilongwe District, Central Malawi [24]. The hospital serves urban and rural populations and provides essential medical laboratory services including DSSM, malaria microscopy, basic haematology and clinical chemistry, blood-grouping and cross-matching and stool and urine analysis. At the time of the audit the laboratory was staffed by two professional laboratory workers (one lab technician and one lab assistant), three health surveillance assistants and one cleaner. One health surveillance assistant was responsible for sputum specimen registration, smear preparation and staining (non-microscopy) on five full days per week. One professional laboratory worker was responsible for microscopy on 4.5 days per week, since the week's first smears prepared for microscopy become available around mid-day on a Monday.

Sputum smear microscopy is used to investigate patients with ≥3 weeks cough (TB suspects) and to monitor response to TB treatment (follow-up patients). TB suspects are asked to submit three sputum specimens collected as spot-morning-spot over two days. Follow-up patients are usually monitored during the second and fifth months of treatment by examining two sputum specimens on each occasion. Specimens are submitted to the laboratory from Monday to Friday and records are kept in standardized laboratory registers. Data extracted from the register included the date the sputum was received, sex, patient category (i.e. TB suspect or follow-up), the number of specimens examined and result of smear microscopy. In order to minimize disruption, data were extracted from the most recent laboratory register that was not in daily use. Data were obtained for 24 April - 23 October 2006 (6 months). The data were used to calculate: the numbers, sex and categories of patients investigated; the number of specimens submitted; the number of follow-up patients with positive smears; the number and sex of TB suspects with positive smears; and the number and sex of smear-positive cases as then defined. An international system of laboratory workload measurement (Welcan) was used to estimate the workload and staff requirements of actual practice during the period [25]. The data were then used to model the expected impact of implementing recent WHO policy recommendations and LED-FM on annual case detection and, by reapplication of the Welcan system, on the annual workload in hands-on person-hours. The predicted impact of skill-mix management on laboratory practice was also modelled.

The Welcan system assigns a value to laboratory tests in terms of hands-on staff time required. Hands-on time may be defined as time that a staff member is actively working. Hands-on time does not, for example, include time while smears are being passively stained (eg auramine staining for FM) but does if the stain needs to be heated periodically (eg carbol fuchsin staining for ZN). The Welcan system is based on observation and timing of procedures in routine laboratories and takes account of batching of smears etc. The system assigns a value of 15 minutes to the complete processing of a single sputum specimen using ZN smear microscopy (from specimen receipt to reporting). In the model, 5 of these minutes were assigned to the non-microscopy component of the processing and a maximum of 10 of these minutes would be assigned to the microscopic examination of the ZN smear until the result was declared. All 10 minutes would be required to declare a smear negative, and proportionately less for a 3+, 2+, 1+ and scanty positive smear. The overall workload would thus be affected by the proportion of positive smears and their grades, as these take less time to examine. To estimate the workload more realistically in the model, shortened examination times were be attributed to positive ZN smears (0.5 minutes for “3+”, 1 minute for “2+”, 2.5 minutes for “1+” and 5 minutes for scanty smears). As data on the grades of follow-up patients smears were not collected from the laboratory register, an average of 2.5 minutes microscopy time was attributed for each positive smear of follow-up patients. It is assumed that the 5 minutes non-microscopy component of the Welcan value for ZN smears is fixed as clerking and processing of the samples is not affected by specimen positivity and there is little opportunity for expediting the process.

The Welcan system assigns a value of five minutes to the complete processing of a single sputum specimen using fluorescence microscopy (from specimen receipt to reporting). In the model, 2.5 of these minutes were assigned to the non-microscopy component of the processing and a maximum of 2.5 of these minutes would be assigned to the microscopic examination of the FM smear until the result was declared (i.e. 25% of the recommended 10 minute ZN microscopy time). All 2.5 minutes would be required to declare a smear negative, and proportionately less for a 3+, 2+, 1+ and scanty positive smear. The overall workload would thus be affected by the proportion of positive smears and their grades, as these take less time to examine. To estimate the workload more realistically in the model, shortened examination times were be attributed to positive FM smears (0.25 minutes for “3+”, 0.5 minute for “2+”, 1.5 minutes for “1+” and 2 minutes for scanty smears). As data on the grades of follow-up patients smears were not collected from the laboratory register, an average of 1.5 minutes microscopy time was attributed for each positive smear of follow-up patients.

The number of staff required for the workload arising from the different interventions was then calculated, using reported details of district laboratory working patterns in Malawi [26]. Finally the total workload was apportioned according to the skills required for processing the specimens at different stages. Level I was assigned for the non-microscopy component and Level II for the microscopy component, with Skill Level I activities conducted by Health Surveillance Assistants (secondary school leavers) and Skill Level II by laboratory assistants or technician (minimum of three years training post-secondary school).

### Ethical Approval

Permission for conducting the audit was obtained from the Lilongwe District Health Office and the National Tuberculosis Control Programme (NTP). General measures are provided in all NTP facilities to ensure patient confidentiality. Data collected during this audit did not include any personal identifiers. The Malawi National Health Science Research Committee provides general oversight and approval for collection and use of programmatic data for monitoring and evaluation and improvement of service delivery. The work described in this report was conducted under this arrangement.

## Results

A total of 2449 patients were registered with a median of 86 patients (range 48–178) and 239 (range 140–482) smears examined per week. Of these, 1920 patients were TB suspects and 529 follow ups. In total 6,796 smears, 5,730 (84.3%) from TB suspects and 1,066 (15.7%) from follow-up patients, were examined. Results from thirty smears from TB suspects were not recorded (4 of 128 smear-positive males and 6 of 72 smear-positive females did not submit the third specimen, the remaining 21 missing smears resulted from between 10 and 20 smear-negative TB suspects submitting only 1 or 2 specimens). Eight follow-up patients had 3 smear examinations recorded possibly in error. Five hundred and sixty one (9.7%) of the 5,730 smears from TB suspects and 49 (4.6%) of the 1,066 smears from follow-up patients had ≥1 AFB resulting in 201 (10.5%) and 26 (4.9%) of the TB suspects and follow up patients being smear-positive. A median of 7 (range 3–13) TB suspects and 1 (range 0–3) follow up patient were positive per week and 19 (range 9–38) and 2 (range 0–6) smears from TB suspect and follow up patients were positive each week.

In total, 1099 (57.2%) of the 1920 TB suspects and 322 (60.9%) of the 529 follow up patients were male. Among the TB suspects, 128 (11.6%) of 1099 males and 73 (8.9%) of 819 females had ≥ one smear with ≥1 AFB (p = 0.06), while among follow-ups, 15 (4.7%) of 322 male and 11 (5.3%) of 206 female were positive (p = 0.8). Discrepancies between numbers are accounted for by patients for whom no sex was recorded or whose smears were not graded.

### The Increase in Smear-Positive Case Detection That Might Be Expected to Stem Directly from Adoption of the WHO-Recommended Lowered Threshold for Definition of a Positive Smear and a Smear-Positive Case

The numbers of TB suspects identified as having smear-positive PTB stratified by the case definitions and sex are shown in Table 1. Adoption of the new smear positive case definition could result in up to 82% increases in the number of patients defined as smear positive cases depending on the case definition in current use and the availability of facilities for supplementary investigations such as radiography or TB culture. Total smear positive TB case detection increases from 110 cases to 200 when moving from a case definition based on two smears with a minimum grade of 1+ to a definition based on 1 smear with at least 1 AFB. Case detection among males increased from 67 to 128 and among females from 43 to 72.

**Table 1:** Number of TB suspects (by sex) classified as smear-positive PTB cases using different thresholds for case definition.

		Number of TB suspects with			
	N	At least 2 smears at 1+ grade or above	At least 2 smears with ≥1 AFB/smear	At least 1 smear at 1+ grade or above	At least 1 smear with ≥1 AFB/smear
Male (%)	1099 (57.3%)	67 (6.1%)**	122 (11.1%)	98 (8.9%)*	128 (11.6%)
Female (%)	818 (42.6%)	43 (5.3%)**	68 (8.3%)	58 (7.1%)	72 (8.8%)
Unknown	2 (0.1%)	0 (0%)	0 (0%)	0 (0%)	0 (0%)
Total	1919†	110 (5.7%)**	190 (9.9%)	156 (8.1%)*	200 (10.4%)

* P<0.05 and **p<0.01 when compared to the definition “At least 1 smear with ≥1 AFB/smear”; Chi square for trend, males, p<0.001; females p<0.05.

† sex not known for one patient.

### The Reduction in Laboratory Workload That Might Be Expected from Adopting a Two-Specimen Strategy for Investigating Suspected Pulmonary TB

If the 1,920 TB suspects had submitted three sputum specimens 5,760 smears should had been examined. Investigating TB suspects by examining only 2 specimens would reduce the actual workload from 5730 to 3840 smears. The first smear to be positive was the first spot smear in 125 (98%) of the 128 males and 70 (97%) of the 72 females, the remaining 3 (2%) male and 2 (3%) female patients were identified as smear positive on the morning smear. If only one smear with ≥1 AFB were considered sufficient for diagnosis, 195 of 200 patients, being positive on the first smear, would not need examination of the second smear. This would reduce the workload by a further 195 smears to 3645. In accordance with current recommendations, the 529 follow-up patients would continue to be monitored with two smears regardless of the results of the first smear requiring 1,058 smears. The total number of smears therefore would have been reduced from 6788 to 4703 (a reduction of 31%).

### The Potential for Examining ZN Stained Sputum Smears for the Recommended 10 Minutes (or the FM Equivalent) with the Existing Designated Laboratory Staff

Trained laboratory workers at district hospital level are estimated to work 1240 hours per year (23.8 hours/week) [26]. The numbers of patients, smears, and smear grades during the six month audit period were doubled to approximate one year's work (Table 2). The Welcan values of 15 minutes for ZN and 5 minutes for FM microscopy, with modifications for positive smears based on the grades of positivity, were applied to this workload. The Welcan time was then calculated according to the non-microscopy and microscopy components. The microscopy time was up to the recommended 10 minutes examination time for negative ZN smears, and up to 2.5 minutes examination time for negative FM smears. The Welcan values for the total work times for ZN and FM are given in Table 2. The 3840 TB suspects examined in one year would require 163,950 minutes and the follow ups would require a further 31,005 minutes to process all their specimens using ZN smear microscopy. The total number of hands-on person hours was then calculated for the non-microscopy and microscopy components (Skill Levels I and II) resulting in 3,249 hours for ZN and 1,108 hours for FM. These workloads would require 2.62 full time hand-on equivalent (FTHOE) staff for ZN and 0.89 FTHOE for FM, respectively. In practice this would require 5 to 6 staff to be employed for these tasks since much time spent in the laboratory could not be considered hands-on.

**Table 2:** Current workload based on examination of three specimens from TB suspects; comparison of ZN and FM.

		No smears#	ZN times			FM times		
			Non-microscopy ×5 mins	Microscopy*	Total	Non-microscopy ×2.5 mins	Microscopy**	Total
TB suspects 3 specimens n = 3840	Neg	10,338	51,690	103,380	155,070	25,845	25,845	51,690
	Scanty	420	2,100	2,100	4,200	1,050	840	1,890
	1+	356	1,780	890	2,670	890	534	1,424
	2+	214	1,070	214	1,284	535	107	642
	3+	132	660	66	726	330	33	363
	Subtotal	11,460	57,300	106,650	163,950	28,650	27,359	56,009
Follow-ups n = 1058	Neg	2,018	10,090	20,180	30,270	5,045	5,045	10,090
	Pos	98	490	245	735	245	147	392
	Subtotal	2,116	10,580	20,425	31,005	5,290	5,192	10,482
	Total	13,576						
Workload	No minutes		67,880	127,075	194,955	33,940	32,551	66,491
	Total No hours		1131.3	2117.9	3249.3	565.7	542.5	1,108.2
	Staff FTHOE †		Skill Level I	Skill Level II	All staff	Skill Level I	Skill Level II	All staff
	Total/1240 hrs		0.91	1.71	2.62	0.46	0.44	0.89

* Time taken to find a ZN smear neg  = 10 mins; Scanty  = 5 mins; 1+  = 2.5 mins; 2+  = 1.0 min; 3+  = 0.5 min. FU pos  = 2.5 mins.

** Time taken to find an FM smear neg  = 2.5 mins; Scanty  = 2.0 mins; 1+  = 1.5 mins; 2+  = 0.5; 3+  = 0.25 min. FU pos  = 1.5 mins.

# No of negative smears and positive smears (at different grades) calculated based on proportions of smears that were negative and positive (at different grades) in laboratory register at Bwaila Hospital.

† Staff Full Time (Hands-on) Equivalent based on real working hours reported by Mundy et al. [26].

The workloads were re-calculated for ZN and FM if a two specimen strategy were adopted for the investigation of TB suspects, as shown in Table 3. This strategy would reduce the total number of hours required for the investigation of TB suspects from 163,950 to 109,574 minutes when using ZN and from 56,009 to 37,666 minutes for FM, still requiring a total of 1.89 and 0.64 FTHOE, respectively.

**Table 3:** Predicted reduction in workload through examining only two specimens from TB suspects; comparison of ZN and FM.

		No smears#	ZN times			FM times		
			Non-microscopy ×5 mins	Microscopy*	Total	Non-microscopy ×2.5 mins	Microscopy**	Total
TB suspects 2 specimens n = 3840	Neg	6,900	34,500	69,000	103,500	17,250	17,250	34,500
	Scanty	268	1,340	1,340	2,680	670	536	1,206
	1+	250	1,250	625	1,875	625	375	1,000
	2+	156	780	156	936	390	78	468
	3+	106	530	53	583	265	27	292
	Subtotal	7,680	38,400	71,174	109,574	19,200	18,266	37,466
Follow-ups n = 1058	Neg	2,018	10,090	20,180	30,270	5,045	5,045	10,090
	Pos	98	490	245	735	245	147	392
	Subtotal	2,116	10,580	20,425	31,005	5,290	5,192	10,482
	Total	9,796						
Workload	No minutes		48,980	91,599	140,579	24,490	23,458	47,948
	Total No hours		816.3	1526.7	2343.0	408.2	391.0	799.1
	Staff FTHOE †		Skill Level I	Skill Level II	All staff	Skill Level I	Skill Level II	All Staff
	Total/1240 hrs		0.66	1.23	1.89	0.33	0.32	0.64

* Time taken to find a ZN smear neg  = 10 mins; scanty  = 5 mins; 1+  = 2.5 mins; 2+  = 1.0 min; 3+  = 0.5 min. FU pos  = 2.5 mins.

** Time taken to find an FM smear neg  = 2.5 mins; scanty  = 2.0 mins; 1+  = 1.5 mins; 2+  = 0.5; 3+  = 0.25 min. FU pos  = 1.5 mins.

# No of negative smears and positive smears (at different grades) calculated based on proportions of smears that were negative and positive (at different grades) in laboratory register at Bwaila Hospital.

† Staff Full Time (Hands-on) Equivalent based on real working hours reported by Mundy et al. [26].

Finally, the workloads were re-calculated for ZN and FM assuming the adoption of a two specimen strategy for the investigation of TB suspects and that one positive smear is sufficient to define a smear-positive case, in accordance with the newly recommended definition. In accordance with current recommendations, examination of two smears was still required for follow-up patients regardless of the result of the first smear. The adoption of this approach would result only in a slightly reduced time needed for microscopy since the second specimen from TB suspects would still have been registered and a smear prepared, fixed and stained. Overall, this approach would only reduce the workload marginally for ZN, from 1.89 to 1.87 FTHOE, and not at all for FM (FTHOE remains 0.64).

## Discussion

At the time of this audit, two positive smears were required to define a smear-positive case based on microscopy alone in Malawi and the required grading of the smears was not stated in its national guidelines. A person with a single positive smear could be categorized as a smear-positive case if they had radiographic abnormalities consistent with TB, or had a positive TB culture. TB culture was not routinely available for the diagnosis of new cases and many diagnostic centres did not have ready access to chest radiography. The situation is the same in 2009. This audit indicates that adoption of the revised WHO smear-positive case definition could significantly increase case detection in men and women by up to 28% at centres without access to chest radiography or TB culture depending on the grade of smear currently accepted as positive. In contrast to a prospective study conducted in Kenya, this retrospective analysis found that the increase in case detection may be significantly higher in men than in women [6]. An adequate explanation for the difference in findings between these two studies, conducted in similar epidemiological settings, is not immediately obvious.

This audit also demonstrates that the laboratory evaluated is overwhelmed with requests for sputum smear microscopy relative to its human resources and equipment. Similar smear microscopy workloads and human resource shortages were described from Ntcheu District Hospital in Malawi over 10 years ago. The laboratory workload and available human resources at Bwaila Hospital are likely to be similar to those at other district hospital laboratories in Malawi and neighbouring countries [11], [26]. If a quality smear microscopy service, based on the recommended ZN examination time of up to 10 minutes were to be provided for the current workload, a total of 2.62 FTHOE staff would be required. Calculating the number of hands-on staff required for services does not take into account the large proportions of staff time that are not actively engaged in laboratory tasks. Depending upon the efficiency of time-management in the laboratory, 2.62 FTHOE staff translates practically to the equivalent of 5–6 staff being employed to manage the smear microscopy workload. As 1.71 FTHOE would be required just for the microscopy, 3–4 staff would need to be employed for the ZN microscopy component of the work. Each of these staff members would require a microscope. The microscopy workload at Bwaila Hospital is currently being performed by about 25% of the staff complement required. Since the amount of time spent on the microscopic examination of each smear will necessarily be short, poor sensitivity of DSSM (similar to that seen in Cameroon) can be expected. If the implementation of new policies and/or new technology could allow ZN smears to be examined for up to 10 minutes, or FM slides for up to 2.5 minutes (to cover the equivalent area of a smear), case detection might be expected to increase by 70%. This would be over and above that resulting directly from the adoption of the new case definition since the study in Cameroon used the new definition of a smear positive case [13]. The time required to examine the FM smear until reliably declared negative would be only 24 seconds more than the average routine smear-examination time in the Cameroon study.

Reducing the number of specimens examined in the investigation of TB suspects from three to two reduced the workload by 31%., but it still would require a total of 1.89 FTHOE staff if ZN microscopy was used. This most likely represents 4 staff employed, with 3 staff (each with a microscope) employed for the microscopy component alone (1.23 FTHOE). In all approaches using ZN smear microscopy the time taken for the microscopy component, and the professional laboratory staff (Skill Level II) time required, was around twice the time taken for the non-microscopy component and the general staff (Skill Level I) time. This has implications for service costs.

The introduction of fluorescence microscopy to an approach in which three specimens from TB suspects are examined considerable reduced workload and particularly the time required of Skill Level II (microscopy) staff. A total FTHOE staff equivalent of 0.89 of which 0.44 FTHOE would be Skill Level II probably brings the total employed staff required to around 2–3 staff. The combination of reducing the number of specimens examined in the investigation of TB suspects and the adoption of fluorescence microscopy brings the clearest indication that workload could be reduced to the levels at which the existing staff employed on smear microscopy at Bwaila Hospital could provide a quality smear microscopy service. A total of 0.64 FTHOE staff split equally between Skill Levels I and II (0.32 FTHOE) brings the total employed staff required to two (one at Skill Level 1 and one at Skill Level II).

The combination of adopting the new WHO recommendations on smear microscopy and introducing fluorescence microscopy can be expected to considerably reduce workload and almost double smear-positive case detection. Furthermore this increase in case detection would be achieved with the existing human resources available and minimal additional equipment.

The adoption of the new WHO smear-positive case definition directly increased case detection (at least by ZN), but the obviation of confirmatory smears would have a negligible impact on workload regardless of whether ZN or FM were used.

Interestingly, the laboratory was equipped with a functional conventional fluorescence microscope, which was infrequently used. Only one member of staff was trained to use it. There are many reports of conventional fluorescence microscopes being poorly accepted by laboratory staff in LMICs [14]. The microscopes are often very complex, the mercury vapour lamps have a limited useful life, they generate a lot of heat, and have to be used in a dark room [14]. Furthermore, users often express concerns about the health dangers of the ultra-violet (UV) light emitted [14].

The new LED-based FM systems come as complete microscopes or as adaptors for existing light microscopes [27]–[32]. The diodes have long life-spans and low energy requirements. They are simple to use, do not generate much heat, do not emit UV light, and do not need a dark-room. A number of large-scale trials and demonstration studies of LED-FM systems have recently been completed and results are awaited [23].

A number of challenges need to be overcome before LED-FM can be implemented widely in NTP diagnostic centres, including the large-scale training that would be required. The most challenging obstacle is the need to develop and validate adequate EQA systems for fluoresce microscopy [14]. Once these are overcome however, the implementation of the new WHO smear microscopy policy recommendations together with LED-FM could substantially reduce the workload and increase case detection. Further research including the analysis of health system costs and the likely impact on patient access to treatment is needed to prospectively validate the gains predicted by this model.
